# Atypical Left Ventricular False Chordae Tendineae

**DOI:** 10.14797/mdcvj.1213

**Published:** 2023-03-07

**Authors:** Amr Darwish, Priscilla Wessly, Nadeen Faza

**Affiliations:** 1 Houston Methodist DeBakey Heart & Vascular Center, Houston Methodist Hospital, Houston, Texas, US

**Keywords:** LV false tendon, chordae tendineae, LV false chord, 3-dimensional transesophageal echocardiography, mitraclip

## Abstract

An 81-year-old female patient with a history of severe secondary mitral regurgitation, hypertension, and paroxysmal atrial fibrillation was seen by the valve team to determine candidacy for transcatheter edge-to-edge repair of the mitral valve. Two-dimensional biplane imaging showed a transverse basal left ventricle false tendon attached to papillary muscles. The position was concerning for interference during deployment of the mitral clip.

Left ventricular false chordae tendineae are fibromuscular structures in the ventricular cavity without connection to mitral valve leaflets. Although first described more than 100 years ago, the pathophysiological significance of these structures remains unclear.^1^ Some studies have suggested that false tendons reduce the severity of functional mitral regurgitation by stabilizing the position of the papillary muscles as the left ventricle enlarges.^2^ Transverse false tendons also are associated with early repolarization, which could be a substrate for ventricular arrythmias.^3^

Figure 1 and Videos 1–3 show cardiac images of an 81-year-old female patient with a history of severe secondary mitral regurgitation, hypertension, and paroxysmal atrial fibrillation. She was seen by the valve team to determine candidacy for transcatheter edge-to-edge repair of the mitral valve. Two-dimensional biplane imaging showed a transverse (localized to one zone) basal left ventricle (LV) false tendon attached to papillary muscles. The position was concerning for interference during deployment of the mitral clip.

**Video 1:** Two-dimensional biplane transesophageal echocardiography, two chamber mid-esophageal view, showing transverse chord-like structure close to mitral valve connecting the papillary muscles; see also at https://youtu.be/rBnvKGOCzgg.

**Video 2:** Three-dimensional transesophageal echocardiography showing atypical left ventricular false tendon; see also at https://youtu.be/8ScrmhzvQZo.

**Video 3:** Two-dimensional transesophageal echocardiography showing successful MitraClip implantation with residual mitral regurgitation and the tendon remained intact; see also at https://youtu.be/XPrZcds-wEk.

**Figure 1:**
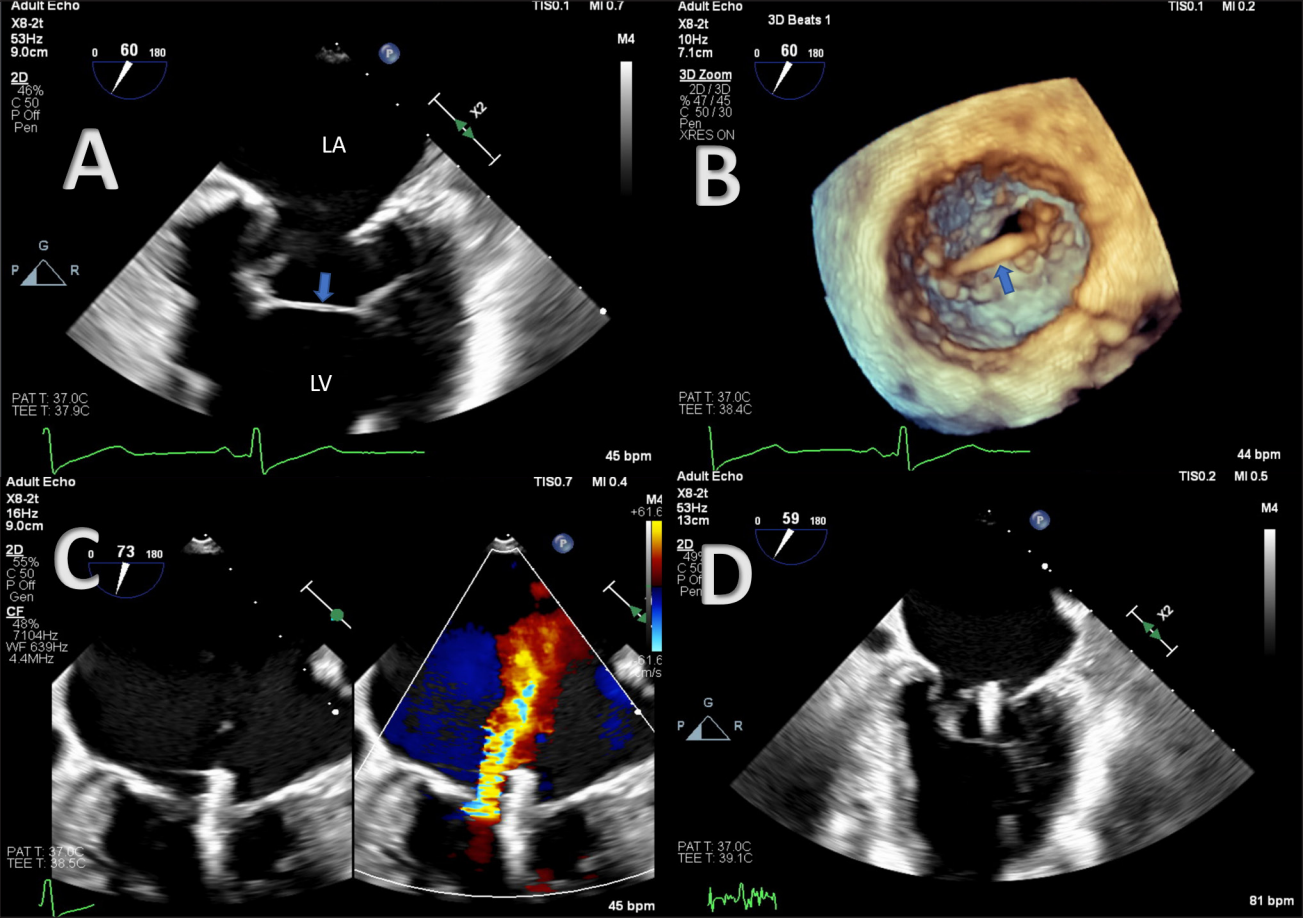
**(A)** Two-dimensional transesophageal echocardiography (TEE) (mid-esophageal 2-chamber view) shows a transverse chord-like structure (blue arrow) connecting the papillary muscles. **(B)** Three-dimensional TEE of the mitral valve highlights the transverse tendon (blue arrow). The procedure was successful with **(C)** residual mild mitral regurgitation, and **(D)** the tendon remained intact. LA: left atrium; LV: left ventricle.
